# Systemic inflammatory indicators and their clinical correlations in prurigo nodularis: A multicenter cross-sectional study in China

**DOI:** 10.1016/j.jdin.2026.05.014

**Published:** 2026-05-25

**Authors:** Xinming Mai, Jiali Wu, Haiyan Huang, Yan Liao, Mengting Yin, Lu Wei, Chener Yang, Jie Zhang, Xia Dou

**Affiliations:** a Department of Dermatology, Peking University Shenzhen Hospital, Shenzhen, China; b Shenzhen University Medical School, Shenzhen, China; c Shenzhen Key Laboratory for Translational Medicine of Dermatology, Institute of Dermatology, Shenzhen Peking University-the Hong Kong University of Science and Technology Medical Center, Shenzhen, China

**Keywords:** atopic dermatitis, clinical characteristics, epidemiology, prurigo nodularis, systemic inflammatory indicators, treatment response

*To the Editor:* Prurigo nodularis (PN) is a chronic inflammatory skin disease characterized by intense pruritus and hyperkeratotic nodules.^1^ Increasing evidence supports PN as a systemic inflammatory disorder rather than a purely cutaneous condition, with associations with atopic diseases, systemic diseases, and neuropsychological comorbidities.^2^ However, simple and accessible biomarkers reflecting systemic inflammation in PN remain poorly defined.

This multicenter cross-sectional study enrolled 390 patients with PN and 378 patients with AD from the China Type II Inflammatory Skin Disease Project (NCT05316805), along with 400 age- and sex-matched healthy controls from the 2015 China Health and Retirement Longitudinal Study. PN was diagnosed according to criteria of the International Forum for the Study of ltch,^3^ with atopic PN defined by the presence of prior or concurrent atopic comorbidities. AD was diagnosed using the Hannifin-Rajka criteria. Clinical severity and patient-reported outcomes were assessed using Investigator Global Assessment, Peak Pruritus Numerical Rating Scale, Dermatology Life Quality Index, and Hospital Anxiety and Depression Scale. The following 6 complete blood count-derived systemic inflammatory indicators were calculated: neutrophil-to-lymphocyte ratio (NLR), monocyte-to-lymphocyte ratio (MLR), eosinophil-to-lymphocyte ratio (ELR), basophil-to-lymphocyte ratio (BLR), platelet-to-lymphocyte ratio (PLR), and systemic immune-inflammation index (SII). Group comparisons and correlations with disease severity were analyzed using standard statistical methods, with *P* < .05 considered significant.

All 6 inflammatory indicators were significantly higher in PN patients than in healthy controls (all *P* < .001). Compared with AD, PN exhibited higher NLR, MLR, BLR, PLR and SII, but lower ELR (Supplementary Table I, available via Mendeley at https://data.mendeley.com/datasets/cpchbn32f8/1). In PN patients, NLR, MLR, PLR, BLR, and SII showed weak but significant positive correlations with Investigator Global Assessment score (R^2^ range 0.025-0.045, all *P* < .05), while ELR was specifically correlated with Peak Pruritus Numerical Rating Scale (R^2^ = 0.118, *P* = .028) (Supplementary Fig 1, available via Mendeley at https://data.mendeley.com/datasets/cpchbn32f8/1). Patients with PN were stratified into high- and low-indicator level groups (above vs ≤median). Those in the higher level group exhibited significantly worse Investigator Global Assessment, Peak Pruritus Numerical Rating Scale, Hospital Anxiety and Depression Scale, and Dermatology Life Quality Index scores, with the strongest associations observed for NLR and BLR (Fig 1 and Supplementary Fig 2, available via Mendeley at https://data.mendeley.com/datasets/cpchbn32f8/1). Subgroup analysis revealed that nonatopic PN primarily differed from AD in NLR, MLR, ELR, and SII (all *P* < .01), whereas atopic PN differed from nonatopic PN in BLR and PLR (*P* < .001) (Fig 2). In a longitudinal subset of 23 PN patients, clinical improvement after 12 weeks of systemic treatment (primarily dupilumab [*n* = 12], JAK inhibitors [*n* = 7], or methotrexate plus compound glycyrrhizin [*n* = 4]) was accompanied by significant reductions in NLR, MLR, and SII (Supplementary Fig 3 and Table II, available via Mendeley at https://data.mendeley.com/datasets/cpchbn32f8/1).

**Fig 1:**
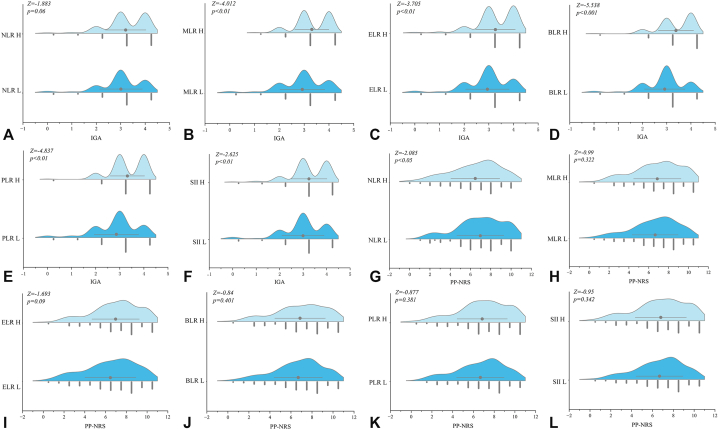
IGA and PP-NRS stratified by high-vs low-levels of inflammatory indices. **A-F,** IGA and **(G-L)** PP-NRS scores across median-stratified NLR, MLR, ELR, BLR, PLR, and SII groups. Notes: Z: Paired sample rank sum test. *BLR*, Basophil granulocyte-to-lymphocyte ratio; *ELR*, eosinophil-to-lymphocyte ratio; *IGA*, Investigator Global Assessment; *MLR*, monocyte-to-lymphocyte ratio; *NLR*, neutrophil-to-lymphocyte ratio; *PLR*, platelet-to-lymphocyte ratio; *PP-NRS*, Peak Pruritus Numerical Rating Scale; *SII*, systemic immune-inflammation index.

**Fig 2:**
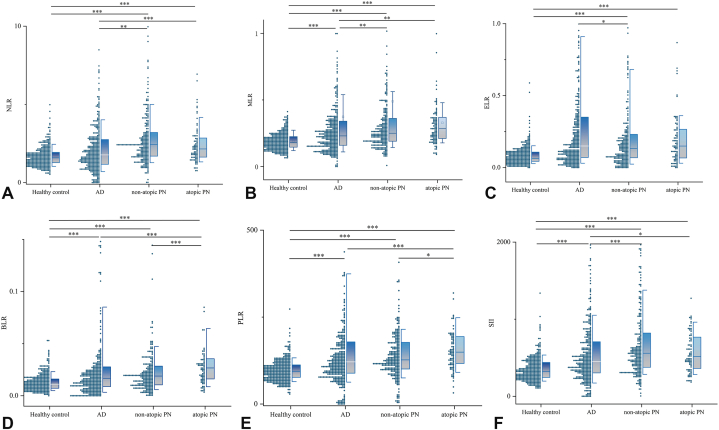
Comparison of CBC-derived inflammatory indices across healthy controls, AD, and PN subgroups. **A-F,** NLR, MLR, ELR, BLR, PLR, and SII levels in healthy controls, AD, nonatopic PN, and atopic PN. Notes: One-way analysis of variance. ∗<0.05, ∗∗<0.01, ∗∗∗<0.001. *BLR*, Basophil granulocyte-to-lymphocyte ratio; *ELR*, eosinophil-to-lymphocyte ratio; *MLR*, monocyte-to-lymphocyte ratio; *NLR*, neutrophil-to-lymphocyte ratio, *PLR*, platelet-to-lymphocyte ratio; *SII*, systemic immune-inflammation index.

Collectively, these findings identify CBC-derived indicators as practical markers of systemic inflammation in PN. NLR, MLR, and SII reflect disease severity and can be used to monitor treatment response, whereas ELR appears to be a marker of pruritus. The neutrophil/monocyte-predominant profile observed in PN contrasts with the eosinophil-dominant pattern in AD, supporting a mixed inflammatory signature in PN.^4^^,^^5^ Importantly, our data highlight immunologically distinct endotypes within PN: nonatopic PN is characterized by neutrophil/monocyte-predominant inflammation, while atopic PN shares features with AD. Further large-scale prospective studies are warranted to validate these observations and to integrate CBC-derived markers into PN clinical decision-making frameworks.

## Conflicts of interest

None disclosed.
